# Advances in Oligonucleotide Aptamers for NSCLC Targeting

**DOI:** 10.3390/ijms21176075

**Published:** 2020-08-23

**Authors:** Deborah Rotoli, Laura Santana-Viera, Maria L. Ibba, Carla L. Esposito, Silvia Catuogno

**Affiliations:** 1 Institute Experimental Endocrinology and Oncology “Gaetano Salvatore” (IEOS), National Research Council (CNR), 80145 Naples, Italy; deborah_rotoli@yahoo.it (D.R.); laura.santana@ieos.cnr.it (L.S.-V.); 2 Department of Molecular Medicine and Medical Biotechnology, “Federico II” University of Naples, 80131 Naples, Italy; m.ibba@studenti.unina.it

**Keywords:** NSCLC, SELEX, aptamer, targeted therapy, diagnosis, biomarker discovery

## Abstract

Non-small-cell lung cancer (NSCLC) is the most common type of lung cancer worldwide, with the highest incidence in developed countries. NSCLC patients often face resistance to currently available therapies, accounting for frequent relapses and poor prognosis. Indeed, despite great recent advancements in the field of NSCLC diagnosis and multimodal therapy, most patients are diagnosed at advanced metastatic stage, with a very low overall survival. Thus, the identification of new effective diagnostic and therapeutic options for NSCLC patients is a crucial challenge in oncology. A promising class of targeting molecules is represented by nucleic-acid aptamers, short single-stranded oligonucleotides that upon folding in particular three dimensional (3D) structures, serve as high affinity ligands towards disease-associated proteins. They are produced in vitro by SELEX (systematic evolution of ligands by exponential enrichment), a combinatorial chemistry procedure, representing an important tool for novel targetable biomarker discovery of both diagnostic and therapeutic interest. Aptamer-based approaches are promising options for NSCLC early diagnosis and targeted therapy and may overcome the key obstacles of currently used therapeutic modalities, such as the high toxicity and patients’ resistance. In this review, we highlight the most important applications of SELEX technology and aptamers for NSCLC handling.

## 1. Introduction

Lung cancer is the leading cause of cancer-related death in the world, with NSCLC representing approximately 85% of all lung cancers [1,2]. In 2018, the World Health Organization counted 2.09 million NSCLC cases and 1.76 million related deaths. Despite great advances in the treatment of NSCLC patients achieved over the past two decades, the overall survival remains low [3]. Indeed, in most cases, patients are diagnosed with advanced or metastatic disease (Stage III or IV), condition for which tumor surgical resection generally cannot be a therapeutic option. In contrast, for patients with Stage I or II NSCLC, surgery represent the main therapeutic strategy, eventually followed by adjuvant chemotherapy [4]. However, some patients may show cancer recurrence even after complete resection. On average the 5-year survival for Stage I and II NSCLC is ~50%. For NSCLC patients at Stage III, platinum-based chemotherapy, usually associated with radiotherapy, represents the main treatment modality [4,5]. After chemo and/or radiotherapy, patients with unresectable tumor can be treated with the antiprogrammed death-ligand 1 (PD–L1) monoclonal antibody (mAb), durvalumab, for immunotherapy. For patients with Stage IV metastatic NSCLC, multimodal chemotherapy administered intravenously in combination with target therapy with the anti-vascular endothelial growth factor (VEGF) mAb, bevacizumab or with immunotherapy with anti-PD–L1 mAb represents the first line strategy. Patients with epidermal growth factor receptor (EGFR), serine/threonine–protein kinase B-raf, anaplastic lymphoma kinase or ROS proto-oncogene 1 mutations can be also treated with specific small inhibitors (afatinib, gefitinib, erlotinib, osimertinib, dabrafenib, crizitinib, alectinib, ceritinib, brigatinib, lorlatinib, crizotinib, entrectinib, cabozantinib). For patients with relapses, the second and the third line treatments depend on the modalities chosen for the first line therapy and on the general patient health conditions and include chemotherapy, immunotherapy and target therapy. Unfortunately, patients with advanced NSCLC (Stage III/IV) show a median overall survival of approximately 10–12 months [4,5,6]. Therefore, a main challenge remains the development of new targeted and combination therapies, as well as the discovery and development of effective tools for early diagnosis, in order to improve patient clinical outcome.

**Figure 1:**
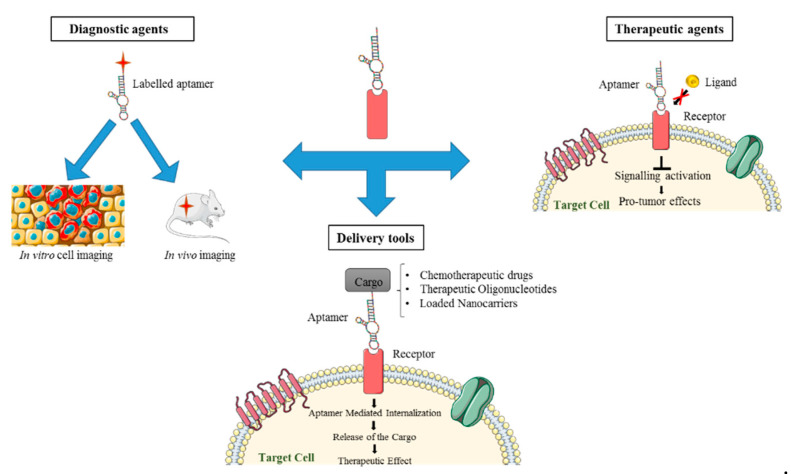
Aptamer biomedical applicability. Aptamers show great potential as diagnostic and therapeutic tools and as delivery carriers of secondary therapeutics.

In this regard, aptamers represent a very interesting class of molecules for various biomedical and pharmaceutical purposes (Figure 1). Aptamers are single-stranded oligonucleotides (DNAs or RNAs) that, by folding into peculiar 3D shapes, bind with high affinity and specificity an explicit target. Because of their mechanism of action, they are also referred to as “chemical antibodies” [7]. Compared to mAbs, aptamers show many useful advantages for biomedical applications. Primarily, they have lower costs, faster and easier procedures of production, and they can be developed against a broad range of targets. In addition, they show improved stability, high batch fidelity and a great plasticity, which render them suitable for different chemical modifications in order to develop them for various clinical uses and to improve their pharmacokinetic and pharmacodynamic properties. Importantly, aptamers show little or no immunogenicity and fewer side effects. Aptamers, besides binding their proper target with high specificity and affinity, are often endowed with inhibitory activity that render them suitable for therapeutic purposes [8]. Moreover, aptamers against cell surface receptors represent very attractive tools for the targeted delivery of different molecules. Indeed, upon binding, aptamers may undergo intracellular receptor-mediated uptake and can drive the specific internalization of secondary reagents of both diagnostic and therapeutic interest [9,10].

Typically, aptamers are produced by a process of combinatorial chemistry known as systematic evolution of ligands by exponential enrichment (SELEX) [11,12] that can be applied to a wide range of targets, from purified proteins to whole living cells or tissues and organisms [13].

In this review, we give an overview of the most important applications of the SELEX technology to NSCLC and provide examples of oligonucleotide aptamer applicability for NSCLC diagnosis and therapy.

## 2. SELEX Technologies to Generate NSCLC Targeting Aptamers

**Figure 2:**
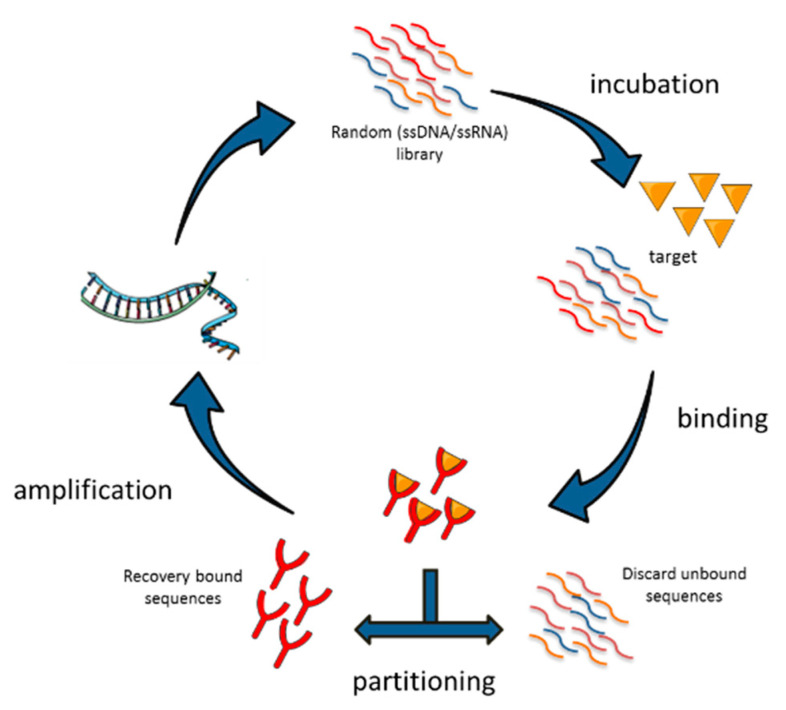
Schematic representation of the systematic evolution of ligands by exponential enrichment (SELEX) procedure. A random single-stranded oligonucleotide library is incubated with the target and then bound sequences are separated from unbound. Bound aptamers are recovered and subjected to amplification. Steps are repeated cyclically to isolate high affinity and specificity aptamers.

Nucleic acid aptamers are selected in vitro by SELEX technology, a multistep process in which a high complexity library of either ssDNA or ssRNAs, undergo repeated rounds of: (i) incubation with the target; (ii) separation of bound oligonucleotides from the unbound fraction; (iii) recovering of bound aptamers and amplification (Figure 2). The reiteration of the cycles allows the progressive enrichment of the library for high affinity ligands. The process generally includes counter-selection steps against unwanted targets in order to improve the aptamer selectivity. To date, many variants of SELEX have been described and applied to a wide range of different targets from purified proteins and small molecules to complex intact cells, tissues or live animals [13]. At the end of the selection, individual sequences are isolated by cloning or sequencing and are screened for the binding affinity and selectivity for the chosen target.

Among the selection strategies, the use of living cells as complex targets may allow the maintenance of the target in a physiological configuration. Further, it may permit to isolate aptamers against a specific cell type, without a prior notion of the recognized epitopes and to obtain multiple molecules able to identify specific cell signatures. Different cell-SELEX protocols have been developed for the targeting of various cancers, including NSCLC (Table 1).

Zhao et al. [14] applied for the first time this kind of selection to NSCLC, addressing the isolation of aptamers specifically recognizing lung adenocarcinoma, the most prevalent NSCLC subtype. To do this, they used NSCLC adenocarcinoma cell line A549 and large cell carcinoma HLAMP cells as target for the selection and counter-selection, respectively. Briefly, they performed 25 rounds in which: 1. A ssDNA library was incubated with the A549 cells (positive selection); 2. Bound sequences were recovered; 3. Bound sequences were incubated with HLAMP cells (counter-selection) to remove oligonucleotides against common epitopes; 4. Unbound sequences were collected and amplified. This strategy led to the isolation of a panel of ssDNA aptamers with improved binding to adenocarcinoma cell lines, than other cancerous lung cells or cancer types.

A variant of this selection has been then developed by Vidic et al. [15] with the final aim to select aptamers against NSCLC circulating tumor cells with the ability to also recognize cancer-stem like cells. The authors used A549 cells as positive target and human blood cells as negative control. After seven SELEX cycles, flow cytometry analyses and sorting were performed to isolate, among the selected sequences, aptamers able to bind A549 cell subpopulation positive to CD90 that has been reported as a marker of stem-like cells [16]. After a deep in silico analysis of the obtained sequences, a high affinity aptamer (A155_18) was identified.

The proposed approach, based on cell sorting coupled to computational analyses, provides an interesting strategy to identify new aptamers for a specific subpopulation. Nevertheless, the in vitro validation of the selected aptamer is restricted to A549 and no data on the recognized molecular target is provided, limiting the evaluation of the real applicability of the isolated sequences.

An additional differential-cell SELEX against A549 cells has been developed by our group [17,18]. In this case, an RNA library modified with 2′fluoro-pyrimidine (2′F-Py) was used to improve the resistance to nuclease digestion. The approach was designed to identify a set of RNA sequences discriminating A549 cells (resistant to TRAIL, cisplatin and paclitaxel) from the more drug-responsive H460 cells. At each round, the library was first incubated on H460 cells for the counter-selection, unbound sequences were recovered and used for the positive selection step on A549 cells. Among the selected sequences, an aptamer binding and inhibiting the EGFR was further characterized as therapeutic tool for NSCLC.

Most of the above described selection approaches, as well as the functional studies mentioned in the following sections, employed A549 cells. This cell line is well characterized and represents the most commonly used model for adenocarcinoma both in vitro and in vivo. However, it should be considered that it does not cover the entire spectrum of NSCLC, i.e., in terms of K-Ras and EGFR mutations. The aptamers obtained on this cell line may thus capture only part of NSCLC (i.e., with KRAS mutant and EGFR wild type).

In order to further enhance the physiological context of the aptamer selection and their applicability, Zamay et al. performed a selection using as positive target lung cells derived from postoperative adenocarcinoma tissues [19]. After a first round consisting of the positive selection step only, the authors performed 10 rounds including prior the selection, two counter-selections on cells from healthy lung and on blood cells from healthy person. The process allowed the successful selection of four aptamers (LC-183, LC17, LC-18, LC-110) and the identification of eight potential lung adenocarcinoma cell protein biomarkers associated, including cathepsin D, vimentin, annexins, histone2B. The selected aptamers did not bind to normal lung cells and lymphocytes, had a very low affinity for A549 cell line and were able to detect circulating tumor cells (CTCs) in patients’ peripheral blood samples.

In addition to differential approaches, whole-living cell-based protocols have been applied by the same group for the selection of aptamers against NSCLC-related known targets [20]. The authors addressed the selection of DNA aptamers against the epithelial cell adhesion molecule (EpCAM), a marker expressed at high levels by many carcinomas as well as by circulating tumor cells [21,22]. They used primary lung cancer cells overexpressing EpCAM as selection target and a competitive displacement with EpCAM specific antibody. Specifically, authors performed seven rounds of *positive selection* by using primary cells from different patient tissues and two additional cycles including antibody displacement steps after the selection. For displacement, the aptamer pool was incubated with receptor positive cells and then EpCAM specific antibody at high concentration was added in order to replace bound aptamers and recover them in solution. The obtained sequences recognize with high affinity EpCAM^+^ lung cancer cells and were successfully used to detect CTCs in liquid biopsies.

Even if cell-based selections permit the preservation of the target structure, they do not mimic at all the in vivo conditions, such as tumor microenvironment influences. To improve the success of the in vivo applicability of aptamers, more recently SELEX protocols have been applied to living animals. Wang et al. described an innovative in vivo selection against NSCLC mice xenografts [23] (Table 1). They used an RNA pool containing 2′F-Py and polyethylene glycol (PEG) modifications to have a good aptamer stability. The selection protocol included 11 rounds of: 1. RNA library injection by tail vein in three NCI-H460 tumor xenograft mice; 2. Sacrifice of the animals after six hours; 3. Recovering of the tumors; 4. Recovering of the bound pool by TRIzol extraction, RNase A and DNase I treatment and amplification.

Among the selected aptamers, a sequence specifically recognizing NCI-H460 tumors, but whose molecular target was not identified, was further characterized as an inhibitor and a delivery carrier of chemotherapeutics in vivo (details are provided in the following sections).

Despite the innovation represented by the in vivo selection approaches, it should be noted that a key aspect is the timeframe chosen for aptamer recovery. Different timing (from 30 min to four hours) has been reported for in vivo SELEX on additional systems [24,25,26], and the parameters behind the chosen time are poorly discussed. In addition, a general consideration on in vivo SELEX is that there are no study comparing this approach with in vitro selections and demonstrating the superior features of in vivo isolated molecules.

## 3. Nucleic Acid Aptamers for NSCLC Detection

As described above, the advances in SELEX technology had allowed the discovery of aptamers that are able to specifically recognize NSCLC cells, thus pointing out their great potential to be used in the field of diagnosis. In this section, we describe several examples of those aptamers that have been exploited for the development of NSCLC detection strategies (Table 2).

First examples are ssDNA aptamers developed by Zhao Z. and colleagues [14], that permitted to discriminate between adenocarcinoma tissue and other lung cancer sections by fluorescence imaging, suggesting their possible exploitation in clinical detection and diagnosis.

Later on, Zhao L. and colleagues used the same ssDNA aptamer pool for the set-up of a microfluidic assay based on a silicon nanowire structure (SiNWS) for the detection and capture of NSCLC CTCs [27]. The clinical relevance of CTCs has been studied for early diagnosis and prognosis of many tumors, including NSCLC [28]. Despite the great progresses in CTC detection, only few methods have entered the clinical practice. Among the limiting factors, there are the CTC low level in the blood stream (approximately one CTC/billion normal hematopoietic cells in the blood of advanced disease patients) that makes necessary to use large blood volumes and CTC heterogeneity [29]. Further, standard methods, employing CTC isolation, enrichment and analyses based on antibodies, result in time-consuming and expensive procedures. In this view, aptamers may provide exquisite and cost-effective recognition elements alternative to antibodies for CTCs capture/detection [30]. Zhao L. et al. took advantage of a previously tested microfluidic assay developed for the capture of CTCs from whole blood samples of NSCLC patients, where conventional antibody-based capture agents were replaced by single aptamers [31]. In the new assay, they used rationally designed cocktails of aptamers that recognize different NSCLC CTC receptors (i.e., different CTCs subtypes), thus improving cell affinity through a synergistic effect. An interesting feature of the system was the possibility to recover the captured CTCs upon treatment with a nuclease solution, allowing the isolation of CTCs for further functional and molecular analyses. Nevertheless, the method showed some limitations in the release of captured CTCs when, instead of artificial spiked samples, real patient blood samples were used, probably because the presence of blood components and to the sticky nanowires utilized [32].

To overcome these disadvantages, the authors have recently developed a new method to exploit the synergistic effect of the aptamer cocktails. The assay used aptamer-coated magnetic beads for the capture of CTCs and a subsequent microwell chip-assisted purification step of the obtained cell suspension [32]. The microwell-assisted multiaptamer immunomagnetic platform (MMAIP) developed was tested for the isolation of CTCs from NSCLC patients’ blood samples collected after chemotherapy treatment. Using modified magnetic nanoparticles with two aptamers (apt1 and apt2) which recognize A549 and cisplatin-resistant A549D cells, respectively, the authors achieved CTC capture and gene analysis from clinical patients’ blood samples [32]. Thus, by means of this noninvasive method, key supplementary information for patient’s diagnosis, prognosis and treatment response, as well as for real time characterization of CTCs was obtained.

The possibility to use aptamers for the detection of CTCs was also described by Zamay et al. [19,20]. They found that fluorescently labeled aptamers selected against lung adenocarcinoma cells from postoperative tissues, when applied to patients’ peripheral blood samples, were able to detect CTCs, with a specificity of 76% and a sensitivity of 86% [19]. More recently, the same authors used two DNA aptamers (ECM-APT-01 and ECM-APT-02) directed against EpCAM to isolate and detect EpCAM^+^ CTCs in clinical blood samples with a detection limit of 0–2 cells/mL of blood [20]. Further, Vidic and colleagues demonstrated that an aptamer (A155_18) isolated by cell-SELEX (see previous section for details) showed a good potential as a diagnostic tool for the detection of NSCLC CTCs expressing the stem cell marker CD90 [15].

Wu and colleagues have instead suggested an alternating aptamer-based approach for in vitro diagnosis of NSCLC [33]. The authors developed what they called a “recognition-before-labeling strategy” for a highly sensitive detection of A549 cells. The assay was based on the use of an aptamer (S11e) isolated and characterized by Zhao and colleagues [14] and of Quantum Dots (QDs) for the labeling. The peculiarity of the method relies on the fact that the aptamer labeling with fluorescent quantum dots (QD) takes place only after aptamer recognition of the target, avoiding the negative effects of the use of pre-labeled DNA aptamers, such as reduced specificity due to size effect and/or steric hindrance.

Later on, the same authors found out that another aptamer (S6) from Zhao et al. [14] folds into a stable G-quadruplex structure in physiological conditions [34] and developed a simple method for the detection of A549 cells. They used the 3,3′-di(3-sulfopropyl)-4,5,4′,5′-dibenzo-9-ethyl-thiacarbocyanine triethylammonium salt dye selectively recognizing G-quadruplex [35]. Their system allowed the detection of lung adenocarcinoma cells in clinical pleural perfusions and in human serum with high specificity and a detection limit of eight cells per milliliter, suggesting potential application in clinical analysis [34].

In addition to the use of labeled aptamers, the development of biosensor based on sandwich systems has been described for NSCLC detection. An interesting example comes from Mir et al. [36]. They developed a cytosensing method by using an aptamer against the protein mucin 1 (MUC1) that is overexpressed in lung cancer. The aptamer was first, covalently immobilized on a conducting polymer nanocomplex formed by the self-assembly of TTBA on AuNPs (apt/TTBA/AuNP) to generate the biorecognition probe used to capture NSCLC cells. Second, the same aptamer was attached with the hydrazine sulfate to AuNPs (Hyd/AuNP/Apt) and incubated with the complex apt/TTBA/AuNP-isolated cells. The binding to the cells of (Hyd/AuNP/Apt) generated amplified electrochemical signals through the hydrazine-mediated reduction of hydrogen peroxide. The authors demonstrated that the developed aptasensor had high affinity and specificity for MUC1^+^ NSCLC cells (A549), discriminating them from MUC1^−^ normal lung cells (MRC-5) or cells from other cancers (human prostate and liver tumors). The system showed a dynamic range from 15 to 1 × 10^6^ cells/mL and a detection limit of eight cells per milliliter.

All the above reports provide important proof-of-concepts about aptamer applicability for CTCs capture and/or detection. The field still requires further studies to evaluate the quantification limit and the detection profile of the proposed approaches in order to define their potential in the clinical practice.

An interesting aptamer-based application for the histochemistry of lung cancer tissues and for the visualization of tumor cells was described by Zamay et al. [37]. Authors characterized three previously selected aptamers (LC-17, LC-18 and LC-224) [19], demonstrating their ability to bind whole lung cancer tissues (tumor cells, connective tissues and blood vessels), likely by recognizing tubulin alpha (LC-17), nuclear lamina and vimentin (LC-18) and actin (LC-224). In addition, they successfully used these molecules for the histochemical analyses of fresh lung adenocarcinoma tissues. Most importantly, violet 650-labeled LC-17 and LC-18 aptamers were applied to the visualization of whole resected unprocessed tumors, demonstrating their potential to be used for intraoperative tumor margin detection.

Finally, innovative multiplexed proteomic platform based on high stable modified aptamers with slow dissociation rates, named slow off-rate modified aptamers (SOMAmers), have been developed and applied to biomarker discovery in several tumors including NSCLC [38]. The technology is able to transform the SOMAmer recognition of target proteins into quantifiable signals and permits to simultaneously measure more than a thousand proteins from different biologic samples. Such strategy has been for instance applied to NSCLC serum biomarker studies (Ostroff et al. 2010a; Mahan 2014), identifying 44 candidate biomarkers and developing a 12-protein panel able to identify NSCLC from controls with high sensitivity and specificity. Further, a 7-protein biomarker (EGFR1, MMP7, CA6, KIT, CRP, C9 and SERPINA3) panel (AptoDetect™-Lung, Aptamer Sciences, Inc., Pohang, Korea) was developed as aptamer-based proteomic technology to distinguish lung cancers from benign nodule controls [39,40].

In addition to the reported examples, there are other promising NSCLC diagnostic markers against which selected aptamers already exist, even if they have not been applied directly to NSCLC. An interesting example is an aptamer against the programmed death-ligand 1 (PD–L1) [41] that has been used for ovarian carcinoma detection. A recent study demonstrated that PD–L1 expression and EMT markers in CTCs negatively correlate with NSCLC patient survival [42], suggesting the diagnostic potential of the anti-PD–L1 aptamer in this tumor type.

## 4. Nucleic Acid Aptamers for NSCLC Therapy

In addition to their high specificity and potential application as diagnostic tools, aptamers are as well promising therapeutic agents. Indeed, aptamer against cancer-related receptors may inhibit the target resulting in an effective antitumor action. To date, several inhibitory aptamers for cancer therapy were characterized and some of them applied for NSCLC treatment (Table 3).

One of the most studied and advanced aptamer in cancer therapy is the AS1411 aptamer, a G-quadruplex molecule targeting nucleolin [43]. Nucleolin is a protein highly expressed on the surface of cancer cells and associated with cell proliferation, angiogenesis and inflammation. In NSCLC, it was shown that cell treatment with AS1411 blocked DNA replication inducing cell cycle arrest in S phase. Studying the mechanism of the anticancer effects of AS1411, Reyes-Reyes et al. [44] found that in A549 cells AS1411 strongly inhibit (~86%) cell proliferation after a 5-day treatment with 10-µM aptamer, as compared with cells treated with the vehicle. This is paradoxically associated with an increase (~80%) in macropinocytosis over baseline levels 48 h after treatment and with Rac1 activation, typically associated with tumor growth promotion. However, different reports indicate that excessive Rac1 activation can instead lead to cell death, through a non-apoptotic mechanism named “methuosis” caused by the hyperstimulation of macropinocytosis [45]. Rac1-induced macropinosomes fuse with each other and with late endosomes to create large vacuoles that lead to cell death by a necrosis-like process, 4–6 days after Rac1 activation [46]. Therefore, the upregulation of nucleolin, limiting Rac1 activation, would represent an adaptive mechanism for cancer cells to avoid methuosis, while the inhibition of nucleolin by AS1411 aptamer can restore methuosis.

Another promising tool for NSCLC targeted therapy is the nuclease resistant 2′F-Py-modified RNA aptamer CL4. The aptamer was isolated by our group through differential cell-SELEX procedure [17] and demonstrated to bind the extracellular domain of the human recombinant EGFR with an apparent Kd of 10 nM, while no appreciable binding was observed on human recombinant ErbB3 extracellular domain. The direct interaction of the CL4 aptamer with the EGFR expressed on cell surface was demonstrated by an aptamer-mediated pull-down assay, as well as by a competition binding assay in the presence of EGF ligand on A549 cells. The specificity of the aptamer on EGFR expressing cells was demonstrated by radioactive-based binding assays on NIH3 T3 cells (EGFR^−^) parental or forced to overexpress the receptor and on A549 cells (EGFR^+^) parental or interfered for EGFR expression. Obtained results demonstrated a preferential aptamer binding on EGFR^+^ cell lines, with a fold increase of ~3 and ~2 (EGFR^+^/EGFR^−^ cells) at 100-nM aptamer treatment, respectively. The aptamer resulted to inhibit EGFR activation in vitro, markedly reducing its phosphorylation (50%, 40% and 70% inhibition at 5, 15 and 30 min of stimulation with EGF), as well as the phosphorylation of the main intracellular effectors ERK1/2 and STAT3. These effects were observed on A549, Calu-1 and A431 EGFR^+^ cells. No inhibitory effects were detected treating cells with a specific scrambled aptamer sequence. The CL4 aptamer, hampering the antiapoptotic STAT3 pathway, inhibited cell viability in vitro of about 60% in A549, Calu-1 and A431 cells following 24 h-treatment, while no effect was observed in H460 EGFR^−^ cells. In A549 target cells the CL4 activated caspases 3, 8 and 9 leading to cell apoptosis, with a percentage of apoptotic cells of about 30% after 24 h-treatment and 40% after 48 h. Further, the aptamer revealed to inhibit tumor growth in vivo in A549-mouse xenografts, leading to a reduction of the tumor mass volume of ~60% following a 16-day treatment (intratumoral injection), as compared with a specific scrambled aptamer sequence. Interestingly, CL4 induced apoptosis also in cells that were resistant to gefitinib and cetuximab, the most commonly used EGFR-inhibitors. These evidences indicate the CL4 aptamer as an effective alternative to the already existing EGFR-inhibitors for NSCLC treatment [17]. Anyway, further investigations are needed to explore its clinical value.

In addition to CL4, our group has applied to NSCLC therapy a 2′F-Py-modified RNA aptamer originally selected against glioma cells [47]. The aptamer, named GL21.T, binds the tyrosine kinase receptor Axl, overexpressed in different tumor types including NSCLC, with high affinity (Kd 13 nM on human recombinant Axl extracellular domain). The aptamer specificity was evaluated by radioactive-based binding assays on different cancer cell lines. The GL21.T aptamer demonstrated to preferentially bind Axl^+^ cells (A549, A431, U87MG, MD-MB-231), rather than Axl^−^ cells (MCF-7 and SkBr3) with a fold increase of ~4. A549 NSCLC cells treated with 400-nM aptamer showed a marked inhibition (~50%) of the Axl receptor phosphorylation induced by 15 min of Gas6 ligand stimulation, leading to a reduced phosphorylation of downstream effectors ERK1/2 and Akt, of ~50% and ~70%, respectively. Comparable results were obtained on U87MG Axl^+^ glioblastoma cells. Despite the inhibition of the ERK1/2 and Akt pathways, the GL21.T aptamer displayed only a poor inhibitory potential on cell viability in vitro (~20%) on the four Axl+ cell lines analyzed (A549, A431, U87MG, MD-MB-231) and no sensitization towards most commonly used chemotherapeutics (cisplatin, paclitaxel and TRAIL) was observed. A strong effect was instead observed on FBS- and Gas6-induced cell migration, with an inhibition of ~60% and ~80%, respectively, as compared with an unrelated aptamer sequence used as a control. In addition, the aptamer showed to reduce the colony formation when cells were grown in semisolid media. The number of spheroids appeared decreased by GL21.T treatment of ~80%. Importantly, the aptamer demonstrated to hamper tumor growth in vivo, in A549-mouse xenografts, leading to a reduction of the tumor mass of ~70% following intratumoral aptamer injection for 22 days [47].

Additional promising targets explored for aptamer-based NSCLC therapy include vimentin and PD–L1/PD-1 networks. vimentin is an intracellular protein found upregulated in lung adenocarcinoma and large cell lung cancer and linked to cancer invasion and metastasis. Zamay at al. [48] described a DNA aptamer (NAS-24) that recognizes vimentin and induces apoptosis in vitro and in vivo. The aptamer was linked to a natural polysaccharide arabinogalactan (NAS–24–AG complex) for its delivery within the cells. The complex or individual moieties were administrated intratumorally for 5 days in Ehrlich ascites adenocarcinoma mouse models, demonstrating that NAS–24–AG complex caused apoptosis in ascites cells more efficiently than free aptamer or AG (percentage of apoptosis of about 20% for AG, 35% for NAS-24 and 50% for NAS–24–AG). The study shows some limits including the lack of experimental negative controls and statistical analyses of the data. However, the interesting point is the use of aptamers against intracellular molecules that underlines the potential aptamer applicability for undruggable targets.

PD–L1/PD-1 network is a key immune checkpoint regulating tumor immuno-escape and its inhibition is one of the most common cancer immunotherapeutic approach. An anti-PDL-1 inhibitory aptamer (aptPD–L1) has been recently applied to lung cancer therapy by Lai et al. [49]. The authors demonstrated that aptPD–L1 inhibited PD–1–PD–L1 interaction. By using LL/2 lung cancer murine syngeneic models, they found that the intraperitoneal administration of the aptPD–L1 led to significant inhibition of tumor growth, reaching ~70% reduction following 20 days. Notably, the aptamer effect was comparable to that obtained with the anti-murine PD–L1 antibody used at doses about eight times higher (1.2 mg/kg aptamer versus 10 mg/kg antibody) Further, looking at the biodistribution, at six hours from the intraperitoneal administration, the aptamer specifically accumulated in PD–L1-expressing tumors, than the other organs or to a control sequence. Even if the study does not compare aptamer versus antibody distribution, the fact that the aptamer gives comparable efficacy at lower doses with respect to the antibody, underlines important advantages of aptamers in terms of cost-effectiveness and safety.

Later on, Ajona et al. explored the combined inhibition of PD-1 signaling with C5a/C5aR1, a complement system contributing to the immunosuppressive microenvironment in lung cancer. The combination strategy included the use of the RMP1–14 antibody blocking PD-1 and an L-aptamer inhibiting C5a signaling [50]. The authors demonstrated that the combined treatment synergistically reduced the tumor growth in lung cancer models, as compared to mice treated with the anti–PD-1 antibody or with the anti-C5a aptamer. In particular, the combined group resulted in a complete tumor rejection (by Day 40) or a tumor-growth inhibition of ~70% (Day 20) in two different syngeneic models. The effects of the combined treatment were associated with the increase of CD8^+^ T cells and IL2. Further, the combination augmented mice survival in lung metastasis models. The study provides a well done preclinical analyses, even if only irrelevant IgG was used as a control, with no mention of a control aptamer and no data on the safety of the combination protocol. Of note, antagonistic aptamers against PD-1 have also been described [51,52] even if, so far, have only been applied to cancers other than NSCLC. Finally, the in vitro-transcribed RNA aptamer (trans-RA16) isolated through in vivo SELEX by Wang and colleagues [23] and its chemically synthesized version (syn-RA16) demonstrated to specifically bind to NCI-H460 NSCLC cells with high affinity, but its molecular target has not been identified [53]. Alexa Fluor 488-labeled aptamers incubated with different cell lines and analyzed at fluorescence microscope demonstrated to bind NCI-H460 cells, while very low or no binding was observed on human lung adenocarcinoma cell line SPC-A1 cells, embryonic kidney HEK293T cells, human cervical carcinoma HeLa cells and human normal lung BEAS-2B cells. No binding was detected using a scrambled RNA aptamer on each cell line. The apparent Kd of the trans-RA16 and syn-RA16 aptamers on NCI-H460 cells were determined by flow cytometry and calculated to be 12.14 ± 1.46 nM and 24.75 ± 2.28 nM, respectively. By immunofluorescence analysis, the Alexa Fluor 488-labeled syn-RA16 aptamer entered into NCI-H460 cells through endocytosis, showing a high colocalization with LysoTracker following two hours and four hours of incubation. The quantity of internalized RNA aptamer during the time has been assessed by qRT-PCR. However, since the assay does not include a step to remove not internalized RNA bound on the cell surface (e.g., high salt washings, proteinase K treatment or RiboShredder RNase Blend treatment), obtained results do not seem to be completely informative. Further, authors analyzed the antiproliferative effect of the trans-RA16 and syn-RA16 aptamers in vitro by cell viability assays on NCI-H460 cells incubated for 48 h with increasing concentrations of the two aptamers. Obtained results demonstrated a similar inhibition rate for both aptamers, with a maximum effect at 300-nM concentration showing ~85% of cell proliferation inhibition. The calculated IC_50_ was 118.4 nM and 105.7 nM for syn-RA16 and trans-RA16, respectively. However, the effect exerted by a nonspecific RNA control aptamer has not been reported. On the other hand, both syn-RA16 and trans-RA16 did not show inhibitory effects on HeLa cells following 48 h treatment at 600-nM aptamer concentration. The biodistribution of aptamers in vivo was analyzed in NCI-H460 tumor-bearing mice injected in the tail vein with Cy5.5-labeled syn-RA16, trans-RA16 or a scrambled aptamer. Accumulation of aptamers in the tumor mass was visible by 30 min to four hours following the injection, with a peak of fluorescence at two hours, while a very low signal was detected for the control aptamer. Quantities of RNAs accumulated in the tumor mass and other organs (lung, heart, liver and kidneys) evaluated by qRT-PCR 3.5 h following aptamer injection indicated that syn-RA16 and trans-RA16 preferentially accumulated in the tumor with a fold increase from 50 to 1000 in respect of other organs, while the quantities of the scrambled aptamer accumulated in the tumor mass were 400–500-fold lower than RA16 aptamers, with no difference in accumulation between the tumor mass and the other organs. The two aptamers showed a similar behavior with the only exception regarding their accumulation in the lung, showing a difference of 2-fold between syn-RA16 and trans-RA16, that authors do not comment on. Further, the S3 truncated version of the RA16 aptamer, including the 5′-end fix region and the 40 central random nucleotides, for a total of 69 nucleotides, resulted to retain a similar binding capacity on NCI-H460 cells, showing a Kd of 63.20 ± 0.91 nM. The S3 aptamer also showed an antiproliferative activity in vitro on NCI-H460 cells, even if to a lower degree if compared with the long version of the aptamer (83 nucleotides), at least under the conditions of time (48 h) and concentration (150 nM) shown. Indeed, S3 inhibited cell growth by 39.32 ± 3.25%, while RA16 by 61.79 ± 3.27%. Therefore, further investigations would be required to verify whether the S3 aptamer retains a similar functional effect to that of the RA16 long aptamer [53].

Interesting aptamers have been also used to enhance NSCLC response to conventional therapies. An example is a DNA aptamer (apMAFG6F), specifically binding to the musculoaponeurotic fibrosarcoma oncogene family protein (MAFG), involved in chemoresistance in NSCLC. The aptamer was selected by protein-SELEX by Vera-Puente O. and colleagues. The authors observed that aptamer transfection induced the rescue of the sensitivity to cisplatin in H23R and H1299 lung cancer resistant cells, with a significant variation of the IC_50_ from 1.52 ± 0.19 to 1.10 ± 0.33 for H23R and from 9.88 ± 0.18 to 6.3 ± 2.45 for H1299. Even if in H1299 cells the aptamer effect was not compared to that of a control oligonucleotide, the experiment in H23R was also performed with other two aptamers selected in the same selection procedure. Since these two aptamers did not result to interfere with cell sensitivity to cisplatin, they can be considered as negative control oligonucleotides, thus supporting the specificity of the aptamer functional effect at least in this cell line. The authors also stated that, after platinum treatment, MAFG6 F aptamer-transfected H23R cells showed a slight (~20%) reduction of MAFG and ROS-detoxifying HMOX1 proteins, but the statistical significance of these results has not been reported. This work represents an initial study, lacking some important experimental controls necessary to precisely define the mechanism of action of the MAFG6 F aptamer.

Beyond the reported examples, many other aptamers selected against epitopes overexpressed on tumor cells have been developed and could be explored for the targeted therapy of NSCLC, provided that the target is expressed on the cell surface [54].

Thus, the use of aptamer therapeutics exhibit a great potential in cancer treatment. Nevertheless, up to date, most the molecules still remain at a preclinical level. A critical issue for aptamer applicability in clinical studies is their poor pharmacokinetics upon systemic administration. Such property may be improved by aptamer chemical modifications, but extensive studies are still lacking.

Interestingly, some authors have recently proposed the development of aptamer-antibody complex as a mean to overcome the therapeutic limitations of both aptamers and antibodies. In NSCLC, Heo et al. [55] investigated the potential of a complex (named oligobody) containing an anti-cotinine antibody and pegaptanib, the aptamer against the neoangiogenic VEGF-165, currently approved for the treatment of the age-related macular degeneration. They performed a preclinical evaluation of the oligobody in A549 cells and A549–xenograft mouse models. The authors found that the complex showed an extended in vivo pharmacokinetics than the aptamer alone and a better tumor penetration than the anti-VEGF antibody bevacizumab. In addition, the oligobody reduced tumor growth upon intraperitoneal injection in A549–xenografts, at similar extent of bevacizumab (>50%). No mouse weight loss or increase of inflammatory cytokines were found upon treatment. The study represents only a proof-of concept and no precise numeric data are provided. However, it proposes an interesting strategy for the development of novel platforms for cancer therapy.

## 5. Aptamer-Based Conjugates for Targeted Delivery in NSCLC

Main limitations of the currently used therapeutic agents for NSCLC treatment include the lack of selectivity for diseased cells, thus reducing their therapeutic index and producing significant toxicity and side effects associated with the nonspecific biodistribution in the body [10]. Therefore, major efforts have been recently focused on the development of targeted cancer therapeutics to circumvent the occurrence of unwanted toxic side effects and to enhance the antitumor activity [57]. In this context, aptamers designed to distinguish between healthy and tumorigenic cells by targeting specific cell surface biomarkers or receptors are especially useful tools. By taking advantage of their high affinity and specificity and their ability to be effectively internalized into target cells, therapeutic compounds or nanocarriers have been conjugated with aptamers for targeted delivery in a cell type-specific manner, thereby improving their local concentration and therapeutic efficacy [9,58]. Key examples of aptamer-based conjugates applied to NSCLC targeting are discussed in the following sections and summarized in Table 4.

The data discussed below clearly demonstrates how the use of aptamers as carriers, facilitates the restriction of the therapeutic effects exclusively to cells positive for aptamer targets, with no action on negative ones. Furthermore, the developed strategies provide elegant technological advances to obtain combined therapies “in one molecule” thus improving the efficacy of the treatment.

Nevertheless, so far the molecular mechanism underlining aptamer-mediated delivery has not been completely understood and studies investigating the fate of the aptamer conjugates and the release of the secondary reagents within the cells are still lacking.

### 5.1. Aptamer-Small Molecule Conjugates

**Figure 3:**
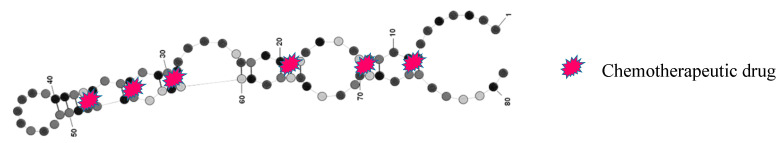
Schematic representation of non-covalent aptamer-drug complexes. Drugs are intercalated within the aptamer sequences.

Aptamers can be used directly to selectively deliver cytotoxic agents to cancer cells. Hu et al. [59] developed an 86-base DNA aptamer (MA3) against a peptide epitope of the MUC1 protein that after doxorubicin (DOX) intercalation (Figure 3) proved, by in vitro experiments, to be capable of carrying the drug and retaining its efficacy into MUC1^+^ A549 tumor cells. A549 cells treated with free DOX or apt-DOX showed a similar viability (78% and 72%, respectively), while HepG2 MUC1^−^ cells treated with free DOX showed a lower viability (68%) if compared with that induced by the treatment with apt-DOX (82%), confirming the specificity of MA3 for MUC1+ cells. Nevertheless, to date, in vivo studies to assess the MA3–DOX complex stability in blood and efficacy have not been published, thus its potential as a therapeutic agent cannot be evaluated at this stage.

Further, Wang et al. generated a non-covalent adduct with epirubicin (EPI) by using the in vivo selected anti-NSCLC inhibitory aptamer (RA16) whose target has not been identified. The authors demonstrated that the PEGylated RA16–EPI molecule gave a higher antitumor effect in NCI-H460 xenograft mice than free EPI or RA16 alone (45.34%, 54.26% and 64.38% inhibition rate, respectively) [23]. However, an important consideration is that recent PEGylated aptamers clinical trials have reported complications that led to premature halt of the trials, due to severe allergic reaction to PEG in few patients (0.6%) and subsequent patient’s death [60,61,62]. Therefore, caution must be taken for the positive evaluation of this aptamer complex in further clinical trials, as well as for other PEGylated aptamers developed. It may be advisable to look for different options to the use of PEG molecules to reduce renal clearance.

### 5.2. Aptamer-Therapeutic RNA Conjugates

Like aptamers, several other types of oligonucleotides, including small interfering RNAs (siRNAs), microRNAs (miRNAs), anti-miRNAs (antimiRs) and small hairpin RNAs (shRNA) are attractive as therapeutic agents because they can modulate the expression of specific cancer targets, including undruggable oncogenes that could not be targeted pharmacologically. However, the efficient and safe delivery of RNAi-based therapeutics into specific tissues or cell populations is still the principal challenge for their clinical development. Thus, the combination of these functional RNAi with oligonucleotide aptamers into a single chimeric molecule has been extensively explored [63,64] and applied to different cancers, including NSCLC.

**Figure 4:**
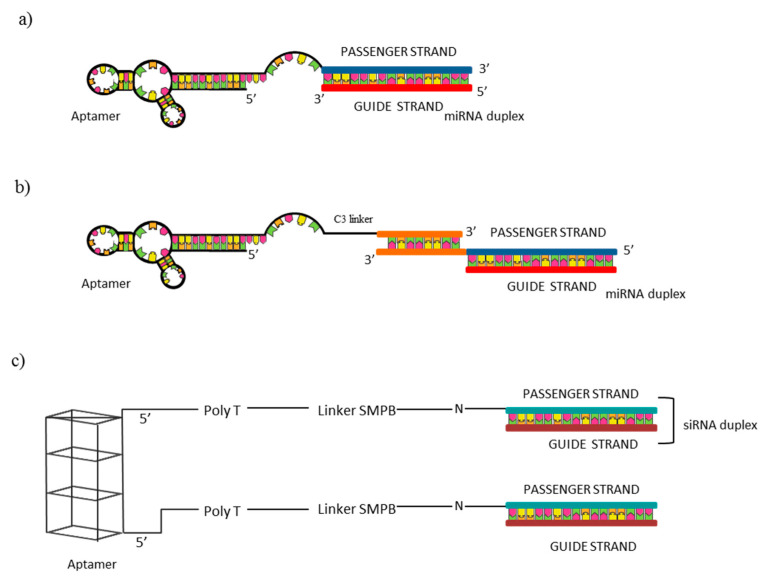
Schematic representation of aptamer–RNA conjugates. (**a**) Conjugation through aptamer extension with the miRNA passenger strand followed by the annealing with the miRNA guide; (**b**) Aptamer and miRNA coupling through the annealing of complementary sticky sequences appended at the 3′-ends of the aptamer and the miRNA passenger strand and subsequent annealing with the miRNA guide; (**c**) aptamer and siRNAs linking by a hetero-bifunctional crosslinker, sulfo-SMPB. A poly(dT) spacer was introduced at the 5′-end to minimize steric interference between the two moieties.

For example, the anti-Axl aptamer GL21.T, selected by our group [47], has been extensively used as a carrier for the selective delivery of miRNAs with tumor suppressor functions in NSCLC. In a first study [65], it was proved that the direct conjugation of let-7g miRNA with the GL21.T was able to retain aptamer binding ability on target A549 (Axl^+^) cells, showing an apparent Kd on human Axl extracellular domain of 19 nM, similar to that of the unconjugated aptamer (13 nM). For the conjugation, a two blocks RNA molecule was generated by extending the aptamer sequence with the miRNA passenger strand, followed by the annealing with the miRNA guide (Figure 4a). Treatment of A549 cells with the 400 nM aptamer-miRNA conjugate resulted in an increase of let-7g miRNA levels of ~12-fold, while no miR upregulation was detected in treated MCF-7 Axl^−^ cells. Interestingly, when MCF-7 cells forced to overexpress Axl were treated with the conjugate a ~9-fold upregulation of the miR was detected, further confirming the Axl-dependent chimera entrance into the cells. Moreover, protein levels of HMGA2, let-7g target, were reduced by ~45% in Axl^+^ cells A similar effect was detected on N-Ras, another predicted target of miR let-7g. Importantly, the treatment of Axl^+^ NSCLC cells with the conjugate resulted in a reduction of A549 cell viability of ~40 % at a concentration rate of 800 nM, while ~20 % of inhibition was reported when cells were treated with the unconjugated aptamer in the same conditions or transfected with 100 nM of a commercial miRNA mimic. The conjugate resulted to inhibit A549 cell migration of ~50%, versus ~30% of inhibition when cells were treated with the unconjugated aptamer or transfected with a commercial miRNA mimic. Finally, the conjugate revealed to decrease tumor growth in mice-bearing A549–Luc xenografts, showing a tumor volume decrease of ~60% following 16 days of conjugate intravenous injection, versus ~45% of tumor volume reduction following unconjugated aptamer injection. Data reported represented the first proof of principle about the possible use of the GL21.T aptamer as a carrier for the selective miRNA delivery in Axl+ cells and demonstrated that the proposed conjugate represent a bifunctional molecule since it combines the antitumor properties of the miRNA and of the aptamer. In another study, by using the same strategy, the GL21.T aptamer was employed for the targeted delivery of the tumor suppressor miR-212 to human NSCLC cells expressing Axl with the aim of restoring TRAIL tumor suppressor pathway [66]. The treatment with the conjugate increased miR-212 in Axl^+^ cells (A549) reaching levels 10-times lower than miR-212-transfected cells but resulting in a comparable PED (miR-212 target) downregulation and functional effects. The inhibition of PED in combination with TRAIL increased apoptosis with a significant caspase 3 activation (about 5–6-fold) than untreated cells. This resulted in reducing cell viability of about 60% in A549 cells. Notably, the conjugate was selective for Axl-expressing cells since no effects were detected in Axl^−^ cells or Axl silenced A549 cells. Functional data were instead confirmed on additional Axl^+^ NSCLC cell lines, Calu-1 and HCC827-ER3, resistant to TRAIL. The study confirms the selectivity of aptamer-mediated delivery, but lacks in vivo data.

More recently, the same aptamer has been used for the delivery of two additional miRNAs, known to be downregulated and to act as tumor suppressors in NSCLC: miR-34c-3p and miR-137 [67,68]. In these cases, the aptamer and the miRNA moieties were coupled through the annealing of complementary sticky sequences appended at the 3′-ends of the aptamer and the miRNA passenger strand, subsequently the miRNA guide strand has been annealed (Figure 4b). The conjugation of the GL21.T aptamer with the above mentioned miRNAs lead to a rapid internalization of the chimera by Axl^+^ NSCLC cells, increasing miRNA cellular levels and downregulating miRNA targets, which in both cases had a negative impact on NSCLC cell migration and growth. In the first study [67], the authors validated the therapeutic potential of miR-34c-3p in NSCLC demonstrating its ability to alter cell viability (50–70% reduction after six days) and colony formation upon its transfection in two cell lines (Calu-1 and A549) than untreated cells or cells transfected with control. Further, it provided evidence that miR-34c-3p regulates Axl translation and enhances the sensitivity to erlotinib in resistant (ER3) HCC827 cells. Then, in order to address miR-34c-3p delivery, the conjugate with GL21.T was generated. Furthermore, in this case, selective binding and miR upregulation upon treatment only in Axl-positive cells was found. Further, the treatment with the conjugate produced the same functional effect of the miR, even if a clear comparison between miR transfection and chimera treatment is not always provided. In the final part, the study addressed an important feature for new therapeutics represented by serum stability and demonstrated that the conjugate is stable up to eight hours in 80% human serum. However, no in vivo data are provided.

The second report [68] characterized the functionality of a conjugate composed by the GL21.T and the miR-137. The most relevant aspect of this molecule relies on the combinatorial therapeutic effects on different cell processes obtained by the two moieties in the complex: the GL21.T aptamer inhibiting Axl receptor and mainly affecting cell migration; the miR-137with an antiproliferative activity. In particular, when applied to Axl-expressing NSCLC cancer cells (A549 and primary NSCLC cultures), the GL21.T-137 conjugate concomitantly inhibited cell migration (of about 40–70%) as GL21.T and reduced cell viability (of about 30–40%) as the miR-137, as well. Further, a first in vivo analyses was provided in A549 tumor-bearing mice. Upon GL21.T or GL21.T-137 intraperitoneal administration (1600 pmol/injection, three times per week) for one week, data showed that the complex resulted in a significant reduction of tumor volumes (about 80% compared to untreated group), further enhancing the extent of inhibition obtained with GL21.T alone (about 50% compared to untreated group). Despite these results are just preliminary, they confirm the potential of GL21.T-137 complex, supporting further analyses in more advanced preclinical settings.

Based on the studies described above, the GL21.T aptamer can be considered an important tool for the selective delivery of secondary reagents to Axl+ cells. Anyway, the mechanism of intracellular processing of the GL21.T-conjugates has not been investigated and further analyses are needed to well understand this point. The use of aptamer–siRNA chimeras with the scope of suppressing tumor invasion and angiogenesis in lung cancer was reported by Wei-Yun Lai et al. [69]. The anti-nucleolin AS1411 aptamer was conjugated with siRNAs against two genes (SLUG and NRP1) and delivered to nucleolin expressing cells. Aptamer and siRNAs were linked together by an hetero–bifunctional crosslinker, sulfo-SMPB. For the conjugation, in the aptamer portion, a poly(dT) spacer was introduced at the 5′-end to minimize steric interference between the two moieties (Figure 4c). Authors observed the effect of single chimeras and combined treatments in nucleolin expressing CL1-5 lung adenocarcinoma cell line. When cells were treated with aptamer–SLUG siRNA or aptamer–NRP1 siRNA as a single treatment, cell migration, analyzed by wound healing assay, was reduced to 59.6% and 39.9%, respectively, if compared with a control chimera in which the AS1411 aptamer was conjugated with a control siRNA. Interestingly, the combined treatment with an half-dose of each aptamer significantly decreased cell migration to 20.6%, indicating a synergistic effect of the two molecules. A similar effect was also observed on cell invasion. Further, results were confirmed in vivo in xenograft mouse models. Following intratumor injection, chimeras reduced tumor growth of ~4-fold if compared with PBS or with a control chimera, but no synergistic effect on tumor growth was observed with combined treatments. The number of CTCs in the blood stream, monitored by qPCR as a measure of cell invasiveness, revealed a significant reduction of CTCs following aptamer–SLUG siRNA and aptamer–NRP1 siRNA injection (~70% and ~80%, respectively) and a synergistic effect was instead detected in this case, with a CTCs reduction of ~90% with the simultaneous injection of the two chimeras. In addition, microvessel formation resulted to be impaired by chimera injection (~60% reduction with single treatment), with a synergistic effect also in this case (~70% with combined treatment).

Therefore, further development and analyses of the combined use of these two aptamer–siRNA chimeras for NSCLC treatment appears very attractive.

### 5.3. Aptamer–Nanomaterial Conjugated Systems

In addition to delivering directly conjugated therapeutic drugs, aptamers can be used to deliver and functionalize nanomaterials, allowing to increase the half-life, the drug payload capacity and the specificity. In addition to their common features, such as biocompatibility for clinical applications, large surface for enhanced aptamer and drug loading and uniform size and shape for excellent biodistribution, nanoparticles (NPs) have other individual physical and chemical properties defined by their materials. However, NPs lack of specificity, therefore, their combination with cell-specific aptamers have been used extensively to develop drug delivery and controlled release systems [63,70].

**Figure 5:**
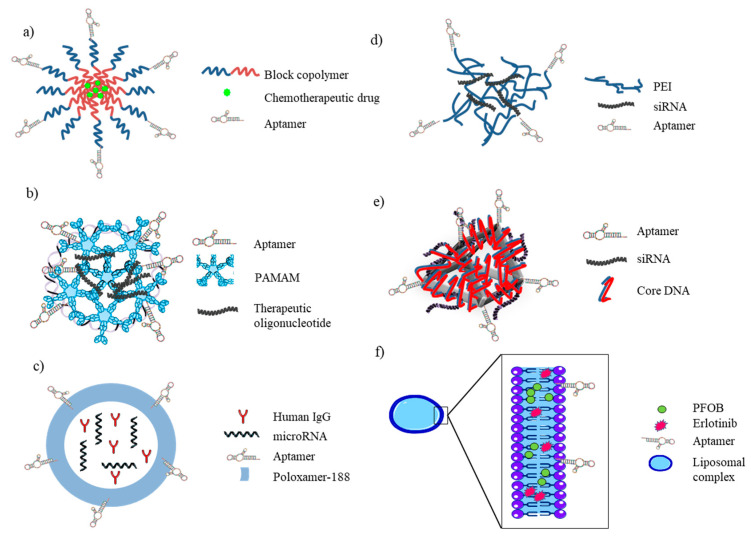
Schematic representation of aptamer–nanomaterial conjugated systems. (**a**) Aptamer–block copolymer; (**b**) aptamer–dendritic structure; (**c**) aptamer–poloxamer; (**d**) aptamer–pDNA/PEI complexes; (**e**) aptamer–DNA nanoprism structure; (**f**) aptamer–liposomal complex.

Polymeric biodegradable NPs have attracted a great interest as effective drug vehicles. Amphiphilic block copolymers, which undergo self-assembly in aqueous solutions to form micelles, represent a well-established approach for the preparation of micellar drug carriers. Many authors have exploited the use of aptamers as targeting moieties of drug loaded block copolymers (Figure 5a), allowing their selective endocytosis. Shira Engelberg et al. used a previously developed DNA aptamer, S15, specifically selected for NSCLC recognition [14] to decorate NPs composed of the biocompatible block-copolymer PEG–PCL entrapping the hydrophobic chemotherapeutic drug paclitaxel (PTX). These NPs demonstrated 2–5 orders of magnitude difference in the selective cytotoxicity towards NSCLCs, pointing out their great potential for selective eradication of human NSCLC cells without harming normal tissues [57]. The mechanism underlying S15 aptamer internalization into human NSCLC A549 cells was previously elucidated by the same authors. Using quantum dots (QDs) decorated with S15 and through the systematic application of a series of established inhibitors of known mechanisms of endocytosis, Shira Engelberg et al. showed that the uptake of the aptamer proceeds via a classical clathrin-dependent receptor-mediated endocytosis. This cancer cell-selective mode of entry could possibly be used in the future to evade an important mechanism of cancer multidrug resistance [71].

Another of the aptamers selected by Zhao et al. against A549 lung cancer cells, S6, was conjugated by Wang and colleagues [72] with polyamidoamine (PAMAM) through difunctional PEG to create a dendritic structure able to encapsulate and selectively deliver to NSCLC cells miR-34a (Figure 5b), whose role in hindering the proliferation and metastasis of NSCLC through the regulation of oncogenes or tumor suppressors, including p53 and BCL2, had been already highlighted [73]. In this study, the authors demonstrated, by qRT-PCR the upregulation of p53 mRNA (7.93 vs 2.1-fold increase) and the downregulation of BCL2 mRNA (60.8% vs 26.5%) and, by western blot analysis, p53 protein overexpression and BCL-2 protein downregulation in in vitro experiments on A549 cells treated with PAM-Ap/pMiR-34a NPs, compared to PAM/pMIR-34a NPs. Moreover, they also reported a significantly higher inhibition of migration (17.7%), invasion (23.8%) and cell growth (29.8%, 49.7%, 57.5% and 62.4% cell viability after 24 h, 48 h, 72 h and 96 h of transfection, respectively) and induction of apoptosis in lung cancer cells treated with targeted NPs, compared with non-targeted NPs. Nevertheless, since the first publication of this conjugate, in vivo studies to evaluate its potential in lung cancer therapy has not yet been published.

A very interesting approach towards the development of smart NPs has been carried out by Shoyele’s group. The uniqueness of their hybrid nanoparticle delivery system relies on the incorporation of human immunoglobulin G (IgG) as the main encapsulating component, which helps to reduce immunogenic reaction by “deceiving” the body to believe that the nanoparticles are natural components of the blood. The outer layer of these hybrid nanoparticles is composed of poloxamer-188, which helps to prevent engulfment by macrophages during systemic circulation. Finally, the presence of MUC1-aptamer [74], allows for the active targeting of payload to MUC1-expressing cancer cells while avoiding undesirable accumulation in healthy cells (Figure 5c). The authors reported that the system was able to successfully deliver miRNA-29b, a tumor suppressor miRNA aberrantly expressed in NSCLC [75], into the cytosol of cancer cells by escaping endocytic recycling, causing a downregulation of oncoproteins DNMT3b and MCL1 in A549 cells, which lead to an antiproliferative effect. They observed a reduction of the cell viability of ~50% if compared to the effect induced by the treatment with MUC1 aptamer-functionalized negative control miRNA-loaded hybrid nanoparticles or by the transfection of the miRNA-29b and an induction of the apoptosis (fold increase of 3.5 and >10 with respect to negative control and lipofectamine 200-transfected miRNA-29b, respectively) [76]. Cell specificity was assessed for NSCLC lines, but not for normal lung fibroblast cell line MRC-5. The nanocomplex firstly accumulated on the outer layer of MUC1^+^ cells and was then internalized via endolysosomal lysis. The internalization of nanoparticles was evaluated using both flow cytometry and fluorescence microscopy analyses. Through in vitro assays, the authors observed a limitation in the release efficiency at pH values of 6.6 and 7.4, thus possibly limiting its release extracellularly in the blood and tumor microenvironment. Nevertheless, optimal release of the payload was obtained at a pH value of 5.0, such as that present in the luminal environment of endosomes/lysosomes. The therapeutic efficacy and pharmacokinetics of the hybrid nanoparticles were then evaluated in lung tumor-bearing SCID mice, showing a 3-fold enhancement of the selective delivery of miRNA-29b to lung tumor cells and inhibition of tumor growth in mouse models, compared to non-functionalized NPs [77]. The authors further proved the potential of their hybrid NP as a treatment modality for NSCLC by encapsulating miRNA-29b and genistein, allowing for the payloads to attack multiple targets. The anticancer effect of these nanoparticles was tested on A549 cells, showing a downregulation of pAKT, p-PI3 K, DNMT3B and MCL1 and demonstrating a superior antiproliferative effect compared to individual genistein and miRNA-29b-loaded nanoparticles (~10%, ~25% and ~60% of cell viability, respectively) [78]. The MUC1 aptamer has also been used for tumor-targeted gene delivery in order to overcome the tunable nonspecific binding of pDNA/PEI complexes (widely used in the field of gene delivery) to negatively charged proteoglycans on cell membranes. The pDNA/PEI/MUC1 aptamer complex (Figure 5d) showed a markedly high gene expression of the luciferase carried by the plasmid in MUC1^+^ A549 cells both in vitro and in vivo, compared to pDNA/PEI or pDNA/PEI/nonspecific aptamer complex. Indeed, the authors reported that in vitro experiments on A549 cells treated with pDNA/PEI, pDNA/PEI/nonspecific aptamer or pDNA/PEI/MUC1 complexes with a weight ratio of 1:1:0.5 (which showed the highest gene expression rate compared to other weight ratios tested) luciferase activity measurements were 6.3 × 10^9^ RLU/mg, 3 × 10^9^ RLU/mg and 17 × 10^9^ RLU/mg, respectively. In in vivo experiments on tumor-bearing mice, the luciferase activity measured were ~1 × 10^9^ RLU/mg, ~0.1 × 10^9^ RLU/mg and ~4 × 10^9^ RLU/mg, respectively [79]. Very recently, the use of MUC1 aptamer was further reported for the development of a new class of drug delivery vehicles for gene therapy based on programmable DNA nanostructures. Liu and colleagues [80] designed and synthesized a DNA nanoprism structure with tunable targeting and siRNA loading capability, composed of MUC1 aptamer and Rab26 siRNA (Figure 5e), and showed, by confocal laser scanner microscopy, that the cellular uptake of the DNA complex was directly proportional to the aptamer number present on each nanoprism structure, which ranges from 0 to 6 aptamer molecule/nanoprism. Moreover, using the DNA nanoprism with three aptamer and 3 Rab26 siRNA molecules, the authors demonstrated, by RTqPCR and MTT assay, the downregulation of Rab26 mRNA expression and the decrease of cell proliferation on two NSCLC cell lines (A549 and H1299) [80].

Further, the AS1411 aptamer has been widely exploited by Mohammad Ramezani’s group for the targeted delivery to NSCLC in three different systems. Two of them were developed aiming at the delivery of shRNA plasmid for specific knockdown of Bcl-xL protein. The first one consisted of a PLL–alkyl–PEI copolymer (as in Figure 5a) [81] while the second used modified PAMAM with 10-bromodecanoic acid (10C) and 10C-PEG (as in Figure 5b) [82]. Both systems were decorated with AS1411 aptamer for targeting nucleolin on target cancer cells. Efficient downregulation of Bcl-xL expression and late apoptosis induction in target cancer cells was reported for both delivery systems with similar ranges (reduction of ~30% of Bcl-xL protein expression and ~11% of late apoptosis induction). The same aptamer was used for the development of a polyethylene glycolepoly (lactic-co-glycolic acid) nanopolymersome loaded with gemcitabine (GEM) (Figure 5a). Compared to GEM-loaded polymersome control, the delivery of the Apt–GEM–NP conjugate to A549 NSCLC cells resulted in enhanced cellular uptake (45% vs. 81%, respectively), cytotoxicity (IC_50_: 28.9 vs. 4.9, respectively) and cell proliferation inhibitory effect, while the aptamer complex showed no efficacy against the free aptamer-treated A549 cells and nucleolin-negative CHO cells [83].

In order to overcome hypoxia-induced drug resistance in lung cancer, Fengqiao Li et al. opted for the use of anti-EGFR aptamer [84] to fabricate a multifunctional liposomal complex to co-administrate erlotinib (an EGFR-tyrosine kinase inhibitor) and PFOB (a type of perfluorocarbon (PFC) widely used as a blood substitute for supplying oxygen to the body) to EGFR-overexpressing NSCLC cells (Figure 5f). Specificity of target recognition and binding efficiency were tested by flow cytometry and confocal imaging using A549, H1975, PC-9 as EGFR^+^ cell lines and EGFR^−^ Helf cells as a negative control. Results obtained by flow cytometry and confocal imaging showed that the entrapped PFOB in the nanoparticle facilitated its uptake compared to nanoparticles loading the drug alone, exhibiting a promising outcome in fighting against hypoxia-evoked erlotinib resistance and in the inhibition of tumor growth, both in vitro and in vivo [85]. Later on, the same aptamer was used again for the delivery of erlotinib, this time in combination with survivin-shRNA, as survivin has been reported to have a crucial role in drug resistance in several cancers [86,87]. The system was developed by using anti-EGFR aptamer (Apt)-modified polyamidoamine in combination with chloroquine (CQ) to normalize tumor vessels for sufficient drug/gene delivery (Figure 5b). Flow cytometry and confocal imaging analysis on PC9 and H1975 EGFR-positive cell lines and on Helf cells as negative control, demonstrated the specificity of target recognition. The authors reported that the use of CQ not only augmented endosomal escape ability, increasing gene transfection efficiency, but also showed a robust vessel-normalization capacity to improve tumor microcirculation, which further increased drug delivery and enhanced drug efficacy in erlotinib-resistant NSCLC cells in vitro, as well as in vivo on H1975 xenograft-mouse models [88].

Furthermore, the use of the anti-EpCAM aptamer [89] as a targeting moiety for the delivery of loaded NPs to NSCLC has been explored both in vitro and in vivo. Alibolandi and colleagues reported that the use of EpCAM-aptamer-decorated, DOX-loaded, PLGA–b–PEG nanopolymersomes (Apt-DOX-NP) (Figure 5a), significantly enhanced cellular nanoparticle uptake in SK-MES-1 and A549 cell lines, as demonstrated by flow cytometry assays (83% and 78% fluorescent intensity increase, respectively) and confirmed by confocal imaging. The authors also reported an increase in the cytotoxicity of the DOX payload. Additionally, Apt-DOX-NP exhibited promising efficacy at inhibiting NSCLC-tumor growth in nude mice bearing SK-MES-1 NSCLC xenografts (46% tumor growth inhibition compared to Dox-NP treated mice and 62% increased inhibition compared to saline treated mice) [90,91].

Finally, the targeting of lung cancer-initiating cells has been as well addressed as well. Xiaolong Huang et al. aiming at targeting a variety of subsets of cancer-initiating cells, developed CD133 [92] and CD44 [93] aptamer-conjugated nanomicelles loaded with gefitinib (CD133/CD44–NM–Gef) to target CD133^+^ and CD44^+^ lung cancer-initiating cells (Figure 5a). Flow cytometry analysis was used to assess the selective targeting of the aptamer conjugates for CD33^+^ and CD44^+^ cells, in both H446 and A549 cell lines. The authors reported a greater therapeutic efficacy against lung cancer-initiating cells of the double targeted nanomicelles than single-targeted and non-targeted ones, thus becoming the first report on drug delivery via nanomedicines targeted to multiple populations of cancer-initiating cells using aptamers [94].

## 6. Conclusions

Over the past two decades, great progress has been made in the treatment of NSCLC, through the application of multimodal therapeutic approaches and the use of mAbs and small inhibitory molecules for the development of molecular targeted therapy and immunotherapy, in combination with chemo and/or radiation therapy. Despite these advances, NSCLC remains a tumor characterized by high mortality—especially for patients with an advanced or metastatic form at the time of diagnosis [95]. Therefore, continued research into new drugs and combination therapies is required to improve patient clinical outcome. The main limits of the current NSCLC management are represented by the frequent late diagnosis (Stage III/IV) and the patients’ resistance to therapies, as well as the onset of numerous serious adverse effects. In this scenario, the scientific research focused on the development of oligonucleotide aptamers as tools for the diagnosis and therapy of NSCLC has achieved very encouraging results on their successful application to overcome these hurdles. Indeed, oligonucleotide aptamers represent a very interesting class of molecules that bind with high affinity and specificity to their targets and can exert a therapeutic effect inhibiting the binding of endogenous ligands. In addition, some aptamers have been demonstrated to internalize in a receptor-mediated manner, thus representing suitable carriers for the targeted delivery of different secondary agents. Moreover, due to their chemical nature, aptamers are not toxic or immunogenic and can be properly modified with the addition of fluorophores or other labels, through the conjugation with nanoparticles, drugs, RNAi or other compounds for both diagnostic and therapeutic purposes. The research works here described show the great potential of aptamer-based approaches in preclinical studies for NSCLC detection and treatment. These noteworthy evidences pave the way for a precision medicine based on the rational design of aptamer-based molecules as a valid option in order to overcome the main obstacles associated with NSCLC treatment modalities.

Despite the great potential of the above described molecules, only the AS1411 aptamer entered a Phase 1 clinical trial in 2006 [96] but did not advance to Phase 2. Thus, the widespread application of aptamers in clinical diagnosis and therapy is still limited.

Part of the reasons for that are independent from aptamers themselves. The process that leads new molecules to invade the clinic is long and passes through long-lasting clinical trials and hundreds of millions of dollars. The delay in clinical advance of aptamers appears less negative, considering that monoclonal antibodies were developed in 1975, but the first antibody-based drug was only approved in 1986 and the second one was not approved until 1994. The process is even more complicated for molecules as the aptamers that overlook a clinical path dominated by strong competitors like antibodies. With this in mind, it is good to hear that the global aptamer market was estimated at 32 million US dollars in 2018 and expected to reach 100 million in 2025—with a CAGR growing rate of 15.5% (https://www.marketstudyreport.com/reports/global-aptamer-market-research-report-2019).

However, it should be noted that several problems related to aptamers still need to be solved for their clinical affirmation. From a therapeutic point of view, key limiting points are aptamer stability and biodistribution upon systemic treatment. The only aptamer approved (the anti VEGFR-165 pegaptanib) is topically used and the success of many aptamer clinical trials was limited by low stability and high accumulation in liver, kidneys and spleen. The aptamer versatility allows to overcome these limitations with the introduction of modifications, but this means that once selected/preclinical evaluated the molecules require improvements.

Another important aspect is the clinical definition of the targets recognized by the aptamers. The development of an aptamer needs to be associated with a clear evaluation of the clinical utility of its target (when and where it is expressed) to speed up the following application of the molecule. On the other end, the possibility to select aptamer against a specific cell phenotype combined with the subsequent target identification is a point of strength of aptamer use and can allow the identification of key signaling pathways that may be then targeted with small molecule drugs. This is for sure a dominant direction of the aptamer field. An additional important trend is the creation of multifunctional molecules, combining aptamers with already-used drugs, including monoclonal antibodies or nanoparticles, in order to obtain improved versions that can more easily enter clinical trials.

The use of aptamers as diagnostics has instead less limitations and risks. In this case, the main problem is the absence of standardized protocols limiting the reproducibility. More efforts need to be devoted to generating unified procedures by employing well-characterized aptamers before developing new detecting molecules.

Despite the problems, the unique features of aptamers are becoming clearer over the years making their extensive use possible in the nearest future.

## Figures and Tables

**Table 1:** Summary of SELEX procedures applied to small-cell lung cancer (NSCLC).

Type of SELEX	Type of Library	Counter-Selection Target	Selection Target	Reference
Differential-cell SELEX	ssDNAs	HLAMP cells	A549 cells	[14]
Differential-cell SELEX	ssDNAs	Human blood cells	CD90^+^ A549 cells	[15]
Differential-cell SELEX	2′F-Py-RNAs	H460 cells	A549 cells	[17]
Differential-cell SELEX	ssDNAs	Cells from healthy lung tissues and blood cells from healthy person	Lung cells derived from postoperative adenocarcinoma tissues	[19]
Competitive-cell SELEX	ssDNAs	–	Primary lung cancer cells overexpressing EpCAM	[20]
In vivo SELEX	2′F-Py-PEG-RNAs	–	NCI-H460 tumor xenograft mice	[23]

**Table 2:** Examples of aptamers used for NSCLC detection.

Aptamer	Target	Reference
Aptamer Pool	NSCLC Subtypes	[14]
S1, S6, S11e, S15	Lung adenocarcinoma cells	
Ap1, Ap2, Ap3, Ap4 aptamer cocktails	NSCLC CTCs	[27]
Ap1–MNP, ap2–MNP cocktail	A549 cells, A549D cells, NSCLC CTCs	[32]
LC-183, LC17, LC-18, LC-110	Lung adenocarcinoma CTCs	[19]
ECM-APT-01 ECM-APT-02	EpCAM^+^ CTCs	[20]
A155_18	CD90^+^ A549 cells	[15]
S11e-QDs	Lung adenocarcinoma cells	[33]
S6–cyM	Lung adenocarcinoma cells	[34]
apt/TTBA/AuNP + Hyd/AuNP/Apt	MUC1^+^ NSCLC cells	[36]

**Table 3:** Examples of aptamers used for NSCLC therapy.

Aptamer	Target	Therapeutic Effect	Reference
AS1411	Nucleolin	Antiproliferative activity in vitro	[44]
CL4	EGFR	Induction of cell death in vitro and tumor growth inhibition in vivo	[17]
GL21.T	Axl	Inhibition of cell viability, migration and colony formation in vitro and tumor growth inhibition in vivo	[47]
NAS-24	vimentin	Induction of apoptosis in vitro and in Ehrlich ascites adenocarcinoma mouse models of the aptamer linked to a natural polysaccharide arabinogalactan	[48]
aptPD–L1	PD–L1	Inhibition of PD–1–PD–L1 interaction and of tumor growth in vivo	[49]
C5a aptamer	C5a	Inhibition of C5a signaling and synergistic reduction of tumor growth and metastasis in combination with anti-PD1 antibody in vivo	[50]
RA16 and its truncated form S3	NSCLC NCI-H460 cells (specific target not been identified)	Inhibition of cancer cell proliferation both in vitro and in vivo	[53]
apMAFG6F	MAFG	Restoration of cisplatin sensitivity	[56]
Pegaptanib	VEGF-165	Reduction of tumor growth with good tumor penetration and extended pharmacokinetics in vivo when complexed to the anti-cotinine antibody	[55]

**Table 4:** Summary of aptamer-based complexes for targeted delivery in NSCLC.

Aptamer	Conjugated System	Cargo	Reference
MUC1	Chemotherapeutic drug	doxorubicin (DOX)	[59]
	Loaded nanoparticle	microRNA-29	[76,77]
		microRNA-29 and genistein	[78]
		pDNA	[79]
		rab26 siRNA	[80]
RA16	Chemotherapeutic drug	epirubicin (EPI)	[23]
GL21.T	Therapeutic oligonucleotide	let-7 g miRNA	[65]
		miR-212	[66]
		miR-137	[62]
		miR-34c	[67]
S15	Loaded nanoparticle	paclitaxel (PTX)	[57]
S6	Loaded nanoparticle	miR-34a	[72]
AS1411	Therapeutic oligonucleotide	SLUGsiR and NRP1siR	[69]
	Loaded nanoparticle	Bcl-xL shRNA	[81,82]
		gemcitabine (GEM)	[83]
anti-EGFR	Loaded nanoparticle	erlotinib & PFOB	[85]
		erlotinib & Survivin shRNA	[88]
anti-EpCAM	Loaded nanoparticle	doxorubicin (DOX)	[90]
CD133 and CD44	Loaded nanoparticle	gefitinib (Gef)	[94]
